# Supplementary material for: Mutations in GDAP1 Influence Structure and Function of the Trans-Golgi Network
Source: Int J Mol Sci. 2021 Jan 18;22(2):914. doi: 10.3390/ijms22020914 (PMC7831947; doi:10.3390/ijms22020914)
Supplement: Supplementary file 1 [file ijms-22-00914-s001.pdf]

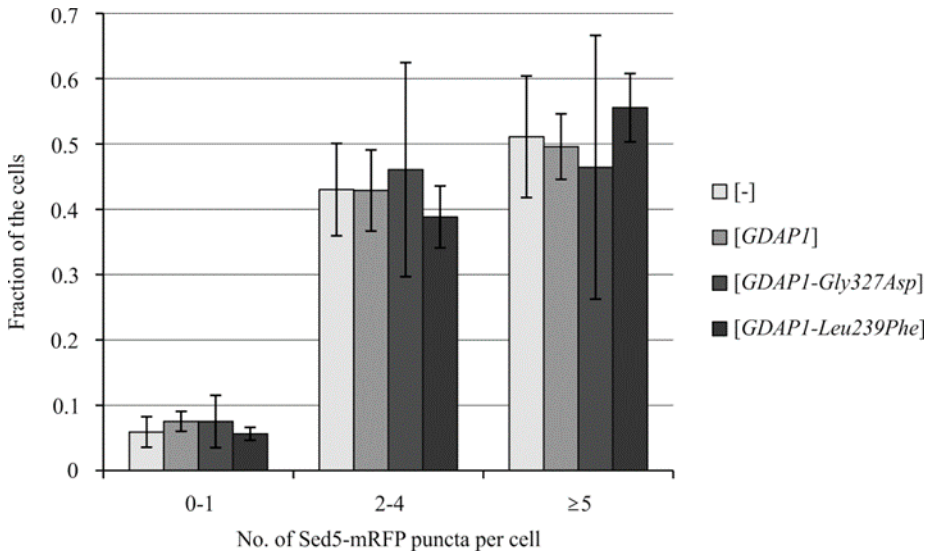

Figure S1. Expression of the *GDAP1* gene variants in yeast cells does not change the localization of the *cis*-Golgi protein Sed5. Yeast bearing the *SED5-mRFP*-containing plasmid (a marker of the *cis*-Golgi network) and indicated plasmids were prepared as in Figure 6. The number of Sed5-mRFP puncta in a single cell was determined. Error bars represent the standard deviation for three repeats.
